# Systematic review of randomized controlled trials for chronic fatigue syndrome/myalgic encephalomyelitis (CFS/ME)

**DOI:** 10.1186/s12967-019-02196-9

**Published:** 2020-01-06

**Authors:** Do-Young Kim, Jin-Seok Lee, Samuel-Young Park, Soo-Jin Kim, Chang-Gue Son

**Affiliations:** 1 Korean Medical College of Daejeon University, 62, Daehak-ro, Dong-gu, Daejeon, Republic of Korea; 2 Institute of Traditional Medicine and Bioscience, Dunsan Oriental Hospital of Daejeon University, 75, Daedeok-daero 176, Seo-gu, Daejeon, Republic of Korea

**Keywords:** Chronic fatigue syndrome, Myalgic encephalomyelitis, RCT, Review

## Abstract

**Background:**

Although medical requirements are urgent, no effective intervention has been proven for chronic fatigue syndrome/myalgic encephalomyelitis (CFS/ME). To facilitate the development of new therapeutics, we systematically reviewed the randomized controlled trials (RCTs) for CFS/ME to date.

**Methods:**

RCTs targeting CFS/ME were surveyed using two electronic databases, PubMed and the Cochrane library, through April 2019. We included only RCTs that targeted fatigue-related symptoms, and we analyzed the data in terms of the characteristics of the participants, case definitions, primary measurements, and interventions with overall outcomes.

**Results:**

Among 513 potentially relevant articles, 55 RCTs met our inclusion criteria; these included 25 RCTs of 22 different pharmacological interventions, 28 RCTs of 18 non-pharmacological interventions and 2 RCTs of combined interventions. These studies accounted for a total of 6316 participants (1568 males and 4748 females, 5859 adults and 457 adolescents). CDC 1994 (Fukuda) criteria were mostly used for case definitions (42 RCTs, 76.4%), and the primary measurement tools included the Checklist Individual Strength (CIS, 36.4%) and the 36-item Short Form health survey (SF-36, 30.9%). Eight interventions showed statistical significance: 3 pharmacological (Staphypan Berna, Poly(I):poly(C_12_U) and CoQ_10_ + NADH) and 5 non-pharmacological therapies (cognitive-behavior-therapy-related treatments, graded-exercise-related therapies, rehabilitation, acupuncture and abdominal tuina). However, there was no definitely effective intervention with coherence and reproducibility.

**Conclusions:**

This systematic review integrates the comprehensive features of previous RCTs for CFS/ME and reflects on their limitations and perspectives in the process of developing new interventions.

## Background

Chronic fatigue syndrome/myalgic encephalomyelitis (CFS/ME) is a long-term debilitating illness characterized by medically unexplained, severe and disabling fatigue that persists at least 6 months and is not improved by rest, accompanied by post exertion malaise (PEM) and unrefreshing sleep [1]. Patients with CFS/ME cannot carry out their normal social routines, work or leisure activities, and some of them are even home- or bed-bound. They experience lower health-related quality of life than those experiencing depression or stroke patients [2]. The medical impact includes the high prevalence in the working age population and particularly the high risk of suicide, which is approximately 7-fold higher than that in healthy controls [3]. An Institute of Medicine (IOM) report in 2015 estimated 17 to 24 billion dollars for total economic costs annually and 836,000 to 2.5 million sufferers of CFS/ME in the USA [1]. The worldwide prevalence of CFS/ME is estimated to be approximately 1–2% [4].

To date, diverse studies, including those of the immune system, metabolomics, endocrine system, gut microbiota and nervous system, have been conducted to determine the pathological mechanisms of CFS/ME [5]. This illness is expected to be a complex, multisystem neuroimmune disease [6]. Recently, some novel clues for CFS/ME were found, such as higher levels of immunosuppressive cytokines, especially TGF-β [7], an altered composition of the gut microbiome [8], and nanoelectronic assays for potential diagnostic biomarkers [9]. However, the clear mechanisms of CFS/ME or its objective diagnostic markers have not yet been found.

In addition, despite numerous approaches with various interventions, no definitively effective treatment has been approved for patients with CFS/ME [10]. Through a large-scale clinical study (called the PACE trial), cognitive behavior therapy (CBT) and graded exercise therapy (GET) were recommended as effective therapies for CFS/ME; however, there is debate and criticism by both scientists and patients [11]. A recent trial using a monoclonal antibody, rituximab, also did not show promising results [12]. At present, the Centers for Disease Control and Prevention (CDC) has proposed symptomatic treatments as an alternative [13]. New approaches and randomized controlled trials (RCTs) are now urgently needed with rigorous experimental designs for therapeutic developments combating CFS/ME.

To facilitate those tasks in the future, this systematic review aimed to integrate the features of the trials for CFS/ME conducted so far in terms of patient characteristics, case criteria, outcome measurements and interventions with overall results.

## Methods

### Data sources and keywords

A systematic literature survey was conducted according to the Preferred Reporting Items for Systematic Reviews and Meta-analysis (PRISMA) guidelines [14] using two electronic literature databases, PubMed (http://www.ncbi.nlm.nih.gov/pubmed) and the Cochrane library (http://www.cochrane.org), through April 2019. The search terms used were encephalomyelitis, ME, chronic fatigue syndrome, CFS, ME/CFS, randomized controlled trial and clinical trial. The trial type was limited to RCTs, and all languages were included.

### Eligibility criteria

Articles were screened according to the following inclusion criteria: (1) RCTs or randomized controlled crossover trials, (2) patients with CFS/ME as participants, (3) an evaluation of the efficacy of the intervention for CFS/ME treatment, and (4) fatigue-related primary measurement or main outcome. The exclusion criteria were as follows: (1) articles with no full text, (2) the number of participants was less than 45 (less than 23 in a crossover trial), (3) studies without mention of the case definition or the characteristics of participants and (4) studies with a Jadad score less than 3 points.

### Data extraction and quality assessment

We extracted data on the number of participants, sex ratio, mean age, ME/CFS diagnostic case definition, intervention category, treatment period, dose, control and outcome measurement tool. We also obtained the outcome data with a statistical analysis of the treatment effectiveness compared to the control.

To assess the quality of RCTs, the Jadad scale was used [15]. The Jadad scale is a five-point scale in which descriptions of randomization, double-blinding, or withdrawals and drop-outs receive one point each. Additionally, a description of the appropriate methods of randomization or blinding receives one point. If the method of randomization or blinding is inappropriate, one point is deducted. Consequentially, trials with ≥ 3 points are considered high quality and were included for further data extraction.

### Judgment of the statistical efficiency of the intervention

We judged the intervention efficacies as ‘Significant’ or ‘Not significant’ based on the data presentations of the original articles. In general, ‘Significant’ meant that the intervention reached statistical significance (intervention vs. control, P < 0.05 or Cohen’s d > 0.8) according to the primary measurement at the planned time point outcome assessment. We defined ‘partially significant’ for the following cases: (1) only part of the main outcomes was statistically significant, or (2) statistical significance was observed only at certain time points without a description of the fixed period for final assessment.

### Data analysis

This study basically does not need to apply statistical analysis. Regarding the number of participants, age and treatment period in two populations (adults and adolescents), data are presented as the mean and standard deviation (SD).

## Results

### Characteristics of RCTs meeting the inclusion criteria

**Fig. 1:**
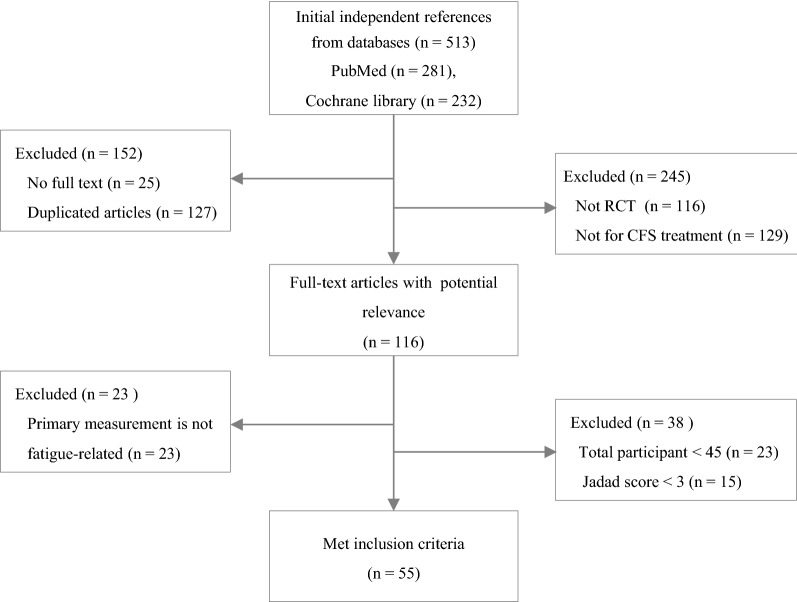
Flow chart of the study

From the PubMed and Cochran databases, a total of 513 articles were initially identified, and 55 articles ultimately met the inclusion criteria for this study (Fig. 1). Fifty RCTs (90.9%) were conducted for adult patients, while 5 RCTs (9.1%) were conducted for the adolescent population (Table 1). The majority of RCTs were conducted in 3 countries: the UK (n = 15), the Netherlands (n = 14), and the USA (n = 9). Regarding interventions, 28 RCTs (50.9%) conducted non-pharmacological interventions, 25 RCTs (45.5%) conducted pharmacological interventions and 2 RCTs conducted a combination of pharmacological and non-pharmacological interventions (Tables 2 and 3).

**Table 1:** Study characteristics

Items	Adults	Adolescents	Total
N. of RCT (%)	50 (90.9)	5 (9.1)	55 (100.0)
N. of participants (%) (males/females)	5859 (92.8) (1466/4393)	457 (7.2) (102/355)	6316 (100.0) (1568/4748)
Mean N. of participants	117.2 ± 87.4	91.4 ± 33.5	114.8 ± 84.0
Mean age (year)^a^	40.3 ± 4.1	15.5 ± 0.3	38.1 ± 8.2
N. of case definitions for inclusion criteria (%)^b,c^			
CDC 1994 (Fukuda)	37 (74.0)	5 (100.0)	42 (76.4)
Schluederberg 1992	2 (4.0)	–	2 (3.6)
Oxford 1991 (Sharpe)	11 (22.0)	1 (20.0)	12 (21.8)
CDC 1988 (Holmes)	3 (6.0)	–	3 (5.5)
Lloyd 1988	2 (4.0)	–	2 (3.6)
Others	5 (10.0)	1 (20.0)	6 (10.9)
RCTs with pharmacological intervention (N, %)	23 (92.0)	2 (8.0)	25 (100.0)
Kinds of interventions (%)	20 (90.9)	2 (9.1)	22 (100.0)
Mean treatment period (weeks)	11.0 ± 7.0	8.5 ± 0.7	10.8 ± 6.8
RCTs with non-pharmacological intervention (N, %)	25 (89.3)	3 (10.7)	28 (100.0)
Kinds of interventions^d^	17 (94.4)	2 (11.1)	18 (100.0)
Mean treatment period (weeks)	16.8 ± 7.2	30.7 ± 15.1	18.3 ± 9.0
RCTs with combined interventions (N, %)	2 (100.0)	–	2 (100.0)
Kinds of interventions (%)	4 (100.0)	–	4 (100.0)
Mean treatment period (weeks)	26 ± 2.8	–	26 ± 2.8
Primary measurements in 55 RCTs (n, %)^c,e^			
Checklist Individual Strength (CIS)		20 (36.4)	
36-item Short Form health survey (SF-36)		17 (30.9)	
Sickness Impact Profile (SIP)		8 (14.5)	
Chalder Fatigue Scale		7 (12.7)	
Visual Analogue Scale (VAS)		6 (10.9)	
Clinical Global Impression (CGI)		5 (9.1)	
Karnofsky Performance Scale (KPS)		3 (5.5)	
School attendance rate (SAR)		3 (5.5)	
Multidimensional Fatigue Inventory (MFI)		2 (3.6)	
Fatigue Severity Scale (FSS)		2 (3.6)	
Others		21 (38.2)	

^a^This is the mean of ages presented as median or mean in original articles

^b^Twelve RCTs used two case definitions for inclusion criteria

^c^Some items have been applied multiple times, thus the total percentage is larger than 100%

^d^One intervention (CBT) was used for both of adult and adolescent studies

^e^Twenty-eight RCTs used multiple primary measurements

**Table 2:** RCTs with pharmacological interventions

Intervention	N. of participants (N. of arms, control)	Dose, period (weeks)	Primary measurement (subscale)	Statistical significance
Psychiatric drugs				
(-)-OSU6162 [16]	62 (2, placebo)	30 mg, 60 mg/day, 2	MFS, CGI	Not significant
Duloxetine [17]	60 (2, placebo)	60–120 mg/day, 12	MFI (general fatigue)	Not significant
Clonidine-hydrochloride [18]	188 (3, placebo, HC)	50 μg or 100 μg/day, 9	Number of steps per day	Not significant
Methylphenidate [19]	60 (crossover, placebo)	10 mg/day, 4	CIS (fatigue, concentration) VAS (fatigue, concentration)	CIS (fatigue): P < 0.01, VAS: P < 0.01
Galantamine hydrobromide [20]	434 (5, placebo)	7.5–30 mg/day, 16	CGI	Not significant
Moclobemide [21]	90 (2, placebo)	450–600 mg/day, 6	Globally improved cases, KPS, POMS	Not significant
Fluoxetine [22]	96 (2, placebo)	20 mg/day, 8	CIS (fatigue)^a^	Not significant
Galantamine hydrobromide [23]	49 (2, placebo)	30 mg/day, 8	VAS (fatigue)	Not significant
Immunomodulators				
BioBran MGN-3 [24]	71 (2, placebo)	6 g/day, 8	Chalder scale(physical)	Not significant
Staphypan Berna [25]	100 (2, placebo)	0.1–1.0 ml/week and 1.0 ml/4 weeks, 24	CGI, CPRS	CGI: P < 0.001, CPRS: P < 0.01
Gamma globulin [26]	71 (2, placebo)	1 gm/kg 3 times/month, 8	Mean functional score	P < 0.05 (6 month)
Poly(I):poly(C_12_U) [27]	92 (2, placebo)	400–800 mg/week, 24	KPS^a^	P < 0.05
Cortisol				
Hydrocortisone + 9-alfa-fludrocortisone [28]	80 (crossover, placebo)	5 mg + 50 μg/day, 12	VAS (fatigue)	Not significant
Fludrocortisone acetate [29]	100 (2, placebo)	0.1 mg/day, 9	Global wellness score	Not significant
Hydrocortisone [30]	32 (crossover, placebo)	5 or 10 mg/day, 4	Chalder scale, CGI	Chalder scale: P < 0.01
Hydrocortisone [31]	70 (2, placebo)	16 mg/m^2^/day, 12	Global wellness score	Not significant
Fludrocortisone acetate [32]	25 (crossover, placebo)	0.1–0.2 mg/day, 6	VAS, SF-36^a^	Not significant
Mitochondrial modulators				
KPAX002 [33]	128 (2, placebo)	12 mg/day, 12	CIS (total score)	Not significant
CoQ_10_ + NADH [34]	73 (2, placebo)	200 mg + 20 mg/day, 8	FIS-40 (total score)	P < 0.05
NADH [35]	26 (crossover, placebo)	10 mg/day, 4	Self-developed subject symptom scoring system	Not significant
Nutrients				
Acclydine [36]	57 (2, placebo)	1000–125 mg/day, 14	CIS (fatigue), SIP-8	Not significant
Polynutrient supplement [37]	63 (2, placebo)	125 ml/day, 10	CIS (fatigue), N of CDC symptoms, SIP-8	Not significant
Others				
Anakinra [38]	50 (2, placebo)	100 mg/day, 4	CIS (fatigue)	Not significant
Ondansetron [39]	67 (2, placebo)	16 mg/day, 10	CIS (fatigue), SIP-8	Not significant
Homeopathic treatment [40]	103 (2, placebo)	Not fixed, 24	MFI	Not significant

*MFS* Mental Fatigue Scale, *CGI* Clinical Global Impression, *MFI* Multidimensional Fatigue Inventory, *CIS* Checklist Individual Strength, *VAS* Visual Analogue Scale, *KPS* Karnofsky Performance Score, *POMS* Profile of Mood States, *CPRS* Comprehensive Psychopathological Rating Scale, *SF-36* 36-item Short Form health survey, *FIS-40* Fatigue Impact Scale-40, *SIP-8* Sickness Impact Profile-8

^a^In cases of no mention for primary measurements or main outcomes in original articles with ≥ 4 measurements, the most fatigue-related measurements were selected by the authors of this review study

**Table 3:** RCTs with non-pharmacological interventions

Intervention	N. of participants (N. of arms, control)	Period (week)	Primary measurement (subscale)	Significance
CBT				
iCBT [41]	240 (3, waitlist)	27	CIS (fatigue)	P < 0.01
Group CBT [42]	204 (3, waitlist)	24	CIS (fatigue), SF-36 (physical score)	CIS: d > 0.8
CBT [43]	122 (2, MRT)	24	CIS (fatigue), SF-36	Not significant
FITNET [44]	135 (2, usual care)	48	SAR, CIS (fatigue), CHQ (physical score)	P < 0.01
CBT + GET [45]	120 (2, usual care)	24	SF-36	Not significant
Family-focused CBT [46]	63 (2, psychoeducation)	24	SAR	Not significant
Group CBT [47]	153 (3, education + support, MC)	16	SF-36 (physical, mental score)	Not significant
CBT [48]	71 (2, waitlist)	20	CIS (fatigue), SF-36 (physical score), SAR	CIS, SF-36: P < 0.01, SAR: P < 0.05
CBT [49]	278 (3, guided support, no treatment)	32	CIS (fatigue), SIP-8	CIS: P < 0.01, SIP: P < 0.05
CBT [50]	60 (2, relaxation)	16–24	Chalder scale, SF-36 (physical score)	Chalder scale: P < 0.01
CBT [51]	60 (2, MC)	16	Karnofsky normal function scale	P < 0.01
Exercise				
Guided exercise self-help [52]	211 (2, MC)	12	Chalder Scale, SF-36 (physical score)	P < 0.01
Qigong [53]	64 (2, waitlist)	16	Chalder Scale, SF-12	Not significant
GET [54]	49 (2, MC)	12	Self-rated global change score	P < 0.05
Education to encourage graded exercise [55]	148 (4, MC)	16	SF-36 (physical score)	P < 0.01
Graded aerobic exercise [56]	66 (crossover, flexibility therapy)	12	CGI	Not significant
Self-care				
Fatigue self-management [57]	137 (3, usual care)	12	FSS	Not significant
Group-based self-management [58]	137 (2, usual care)	16	SF-36 (physical score)	Not significant
Guided self-instruction [59]	123 (2, waitlist)	20	CIS (fatigue), SF-36 (physical, social score)	CIS: P < 0.01
Stepped care [60]	171 (2, CBT)	16	CIS (fatigue), SIP-8, SF-36 (physical score)	Not significant
Guided self-instruction [61]	169 (2, waitlist)	16	CIS (fatigue), SIP-8, SF-36 (physical score)	CIS, SIP8: P < 0.01
Rehabilitation				
Pragmatic rehabilitation [62]	302 (3, supportive listening, general treatment)	18	Chalder scale, SF-36 (physical score)	Not significant
Integrative, consumer-driven rehabilitation [63]	47 (2, delayed program)	16	CFS symptom rating form, the QoL index	P < 0.05
Acupuncture				
Acupuncture [64]	150 (3, sa-am, no treat)	4	FSS	P < 0.05
Acupuncture [65]	100 (2, sham)	4	Chalder Scale, SF-12, GHQ-12 (mental score)	Chalder scale: P < 0.05
Others				
Abdominal tuina [66]	77 (2, acupuncture)	4	Chalder Scale, SAS, HAMD	P < 0.05
Low-sugar, low-yeast diet [67]	52 (2, healthy eating)	24	Chalder scale, SF-36	Not significant
Distant healing [68]	409 (4, not knowing, no treat)	24	SF-36 (mental score)	Not significant

*CBT* cognitive behavior therapy, *FITNET* Fatigue in Teenagers on the interNET, *GET* graded exercise therapy, *CIS* Checklist Individual Strength, *SF-36* 36-item Short Form health survey, *SAR* school attendance rate, *CHQ* Child Health Questionnaire, *SIP-8* Sickness Impact Profile, *CGI* Clinical Global Impression, *FSS* Fatigue Severity Scale, *GHQ-12* General Health Questionnaire-12, *SAS* Self-rating Anxiety Scale, *HAMD* Hamilton rating scale for Depression

### Characteristics of participants and case definitions for inclusion criteria

In 55 RCTs, a total of 6316 participants (1568 males and 4748 females, 5859 adults with a mean age of 40.3 ± 4.1 years and 457 adolescents with a mean age of 15.5 ± 0.3 years) were enrolled. Fifty-four RCTs (98.2%) adapted at least one of the following CFS case definitions: CDC 1994 (Fukuda) criteria (42 RCTs), Oxford 1991 (Sharpe) criteria (12 RCTs), CDC 1988 (Holmes) criteria (3 RCTs), Lloyd 1988 criteria (2 RCTs), and Schluederberg 1992 (2 RCTs). There were 12 RCTs with two case definitions for inclusion criteria (Table 1).

### Main outcome measurement

A total of 31 primary measurement tools were used to assess the main outcome in 55 RCTs. The Checklist Individual Strength (CIS) was the most frequently used (36.4%), and others included the 36-item Short Form health survey (SF-36, 30.9%), Sickness Impact Profile (SIP, 14.5%), Chalder Fatigue Scale (12.7%), Visual Analogue Scale (VAS, 10.9%) and Clinical Global Impression (CGI, 9.1%). There were 28 RCTs that used multiple primary measurements (Table 1).

### RCTs with pharmacological interventions

A total of 22 different medications were evaluated by comparison with placebo in 25 RCTs (23 for adults, 2 for adolescents). These medications included psychiatric drugs (n = 8), cortisol (n = 5), immunomodulators (n = 4), and mitochondrial modulators (n = 3). The mean treatment period was 10.8 ± 6.8 weeks (11.0 ± 7.0 weeks for adults, 8.5 ± 0.7 weeks for adolescents). Three RCTs showed positive results with statistical significance: two with immunomodulators (Staphypan Berna [25] and poly(I):poly(C_12_U) [27]) and one with CoQ_10_ + NADH [34] (Table 2).

### RCTs with non-pharmacological interventions

There were 28 RCTs in the non-pharmacological category (25 for adults, 3 for adolescents) with 18 kinds of interventions, mainly CBT (n = 11), exercise (n = 5), and self-care (n = 5). The mean treatment period was 18.3 ± 9.0 weeks (16.8 ± 7.2 weeks for adults, 30.7 ± 15.1 weeks for adolescents). Of the 11 CBT subcategories, 5 RCTs showed statistical effectiveness of CBT compared to the control [41, 44, 48, 49, 51]. In addition, 3 RCTs of graded-exercise-related therapies [52, 54, 55] and 3 RCTs of integrative, consumer-driven rehabilitation [63], acupuncture [64] and abdominal tuina [66] showed a significantly effect of the intervention compared to the control (Table 3).

### RCTs with pharmacological and non-pharmacological combined interventions

Two RCTs were conducted to assess the synergistic effects of 4 different interventions (GET + fluoxetine, dialyzable leukocyte extract (DLE) + CBT). No synergistic efficacy was observed (Table 4).

**Table 4:** RCTs with pharmacological and non-pharmacological combined interventions

Intervention	Design, N. of participants	Period (week), dose	Primary measurement	Significance
Fluoxetine + graded exercise [69]	Exercise + fluoxetine: 33 Exercise + placebo: 34 Appointment + fluoxetine: 35 Appointment + placebo: 34	24 20 mg/day	Chalder scale	Graded exercise P < 0.05
Dialyzable leukocyte extract (DLE) + CBT [70]	DLE + CBT: 20 DLE + clinic: 26 Placebo + CBT: 21 Placebo + clinic: 23	28 5·10^8^ leukocytes 8 times biweekly	VAS (global well-being)	Not significant

*VAS* Visual Analogue Scale

## Discussion

Since CFS was first shed light on and defined in the 1980s [71], numerous studies on its pathophysiology and treatment have been conducted. Nonetheless, CFS/ME is still poorly understood. To support future studies for CFS/ME treatments, we systematically reviewed 55 RCTs to investigate characteristics such as participants, case definitions, interventions and primary measurements. In addition, we found a trend in the interventions used as well as their overall results.

The sex ratio of the participants was male 1 vs. female 3 (1568/4648, except one RCT had recruited only females). An epidemiological feature of CFS/ME is the higher prevalence in women and even in adolescent populations [72] (Table 1). The diagnostic criteria of the RCTs were diverse. To date, no objective diagnostic parameters or biomarkers exist; thus, the use of criteria for case definitions is the only way to diagnose CFS/ME [73]. Two major case definition tools, Oxford 1991 (Sharpe) and CDC 1994 (Fukuda), have been applied predominantly (Table 1). The former was mostly applied in RCTs conducted before the mid-2000s and preferred by UK studies (10 of the relevant 12 RCTs). On the other hand, CDC 1994 (Fukuda), revised after CDC 1988 (Holmes), has been employed most frequently and steadily by worldwide researchers since 1994.

A total of 55 RCTs included 25 pharmacological, 28 nonpharmacological and 2 combined interventions (Table 1). The mean treatment period of the RCTs with non-pharmacological interventions was longer than that with medication, especially for adolescents (total: 18.3 ± 9.0 vs. 10.8 ± 6.8, adolescent: 30.7 ± 15.1 vs. 8.5 ± 0.7, Table 1). Periodically, the trials gradually increased, with 13 trials in the 1990s, 19 trials in the 2000s and 23 trials in the 2010s. The pharmacological RCTs were predominant in the 1990s and 2000s, while nonpharmacological interventions became predominant in the 2010s (pharmacological:non-pharmacological ratio from 20:14 to 7:16) (data not shown). This trend might be related to the poorly understood etiology of this disease, the knowledge of which is vital for the proper development of therapeutic medications [74].

In the early days, immunological, virological, hypothalamic–pituitary–adrenal (HPA) axis dysfunctional and psychiatric hypotheses were mainly proposed for the pathophysiology of CFS/ME [75]. Accordingly, immunomodulators, cortisol medications, and psychiatric drugs were frequently employed for medication (Table 2). Although some immunomodulators have presented notable positive effects in RCTs [25, 27], they are rarely administered clinically because of potential adverse effects and insufficient evidence of efficacy [76, 77]. Similarly, hydrocortisone or fludrocortisone treatments to modulate the dysfunction of the HPA axis have failed to show the repeatability and coherence of effectiveness [28–32]. Psychiatric drugs, especially antidepressants, have been frequently and steadily employed in RCTs and in the clinical fields [78]. In fact, depressive mood is a common comorbid symptom in CFS/ME patients [79, 80]. However, depression and CFS/ME are well defined as two different diseases. For example, major depressive disorder has a typical pathology of insufficient activity of serotonin (5-HT), while hyperactivity of 5-HT is a feature of CFS/ME [81, 82]. Although there is conflicting evidence, antidepressants are currently not recommended for patients with CFS/ME without depressive symptoms [72]. However, mitochondrial dysfunction and ATP depletion have recently been regarded as weighty features of CFS/ME [5, 83]. Among the two RCTs with those interventions, KPAX002 failed to demonstrate its effects on CFS/ME [33], but NADH + CoQ_10_ showed positive effects on fatigue [34].

Non-pharmacological interventions could be subgrouped into CBT, exercise such as GET, self-instruction with/without guidance, rehabilitation and acupuncture (Table 3). In fact, only CBT and GET were tested for clinical efficacy in CFS/ME in the 1990s. Until 2010, mostly positive outcomes were reported for CBT and GET (3 of 5 RCTs in the 1990s and 4 of 5 RCTs in the 2000s). These RCT-derived results supported and recommended CBT and GET as treatment options for patients suffering from CFS/ME. CBT is a psychosocial therapy that has been applied to diverse mental disorders, including depression, anxiety disorders, personality disorders and psychosis [84–87]. Until 2010, RCTs for CBTs were conducted mostly in the classic form of face-to-face therapy between therapists and patients with CFS/ME [48–51]. Subsequently, various forms of CBT have been employed, such as group CBT [42, 47], internet-based CBT [41, 44], and family-focused CBT [46]. Contrary to the positive outcomes in the 1990s and 2000s, more recent CBT trials have failed to show consistent benefits in patients with CFS/ME: 5 of 7 RCTs of CBT did not show significant effects in our data. Another frequently applied non-pharmacological intervention is GET, a physical activity with a gradual increase in intensity. The hypothesis of GET effectiveness is based on psychiatric assistance through motivating patients to overcome their negative perceptions as well as through an intensification of physical fitness [54]. In our data, 4 of 5 RCTs with graded-exercise-related therapies presented positive outcomes; however, the clinical usefulness of GET is highly controversial [88]. One survey reported that 79% of CFS/ME participants felt that GET worsened their health status [89]. Furthermore, GET was criticized due to the conflict with PEM, a particularly essential symptom of CFS/ME according to the IOM diagnostic criteria [90]. Both CBT and GET have limitations and have received criticism more recently because they are based on psychiatric views, which is contrary to the fact that CFS/ME is a physical illness based on accumulated evidence from scientists as well as patients and physicians [1].

RCTs of alternative medicines and self-therapies for CFS/ME have increased since the late 2000s. Regarding RCTs of alternative medicines such as acupuncture, qigong, and abdominal tuina [53, 64–66], 10 RCTs were selected in our review process, but 6 were excluded due to low quality (a Jadad score less than 3) or too few participants. A systematic review also presented the limitations of acupuncture treatment due to the low quality of RCTs and the weak strength of evidence along with the weak potential to improve the symptoms of CFS/ME [91]. There were also 5 RCTs with self-care therapies, including guided self-instructions and fatigue self-management (Table 3). Among them, 2 guided self-instructions, using a similar protocol of CBT, presented partially positive results [57, 59]. For psychiatric disorders, the therapeutic relationship between patients and therapists is known to play an important role in counseling treatments, and these positive outcomes from interventions with minimized involvement of therapists support an assertion of the nonpsychological aspect of CFS/ME [92].

**Fig. 2:**
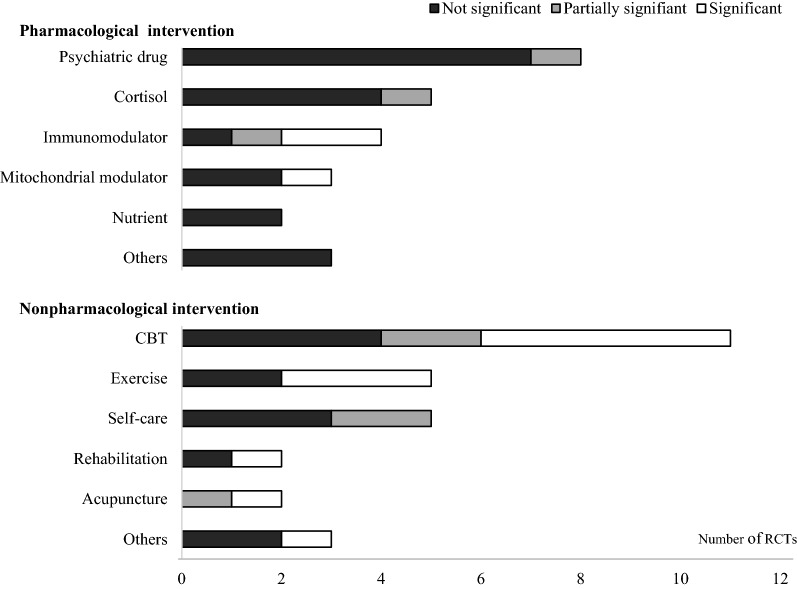
Graphical display for statistical significance of interventions. ‘Significant’ means that the treatment achieved statistical significance (intervention vs. control, P < 0.05 or Cohen’s d > 0.8) according to the primary measurement at the planned time point outcome assessment. ‘Partially significant’ means (1) only the part of the main outcomes was statistically significant or (2) statistical significance was observed only at certain time points without a description of the fixed period for final assessment

To date, the overall results of RCTs have been more positive for non-pharmacological interventions than for medications (Fig. 2). Among diverse interventions, psychiatric approaches were predominant in both pharmacological interventions and non-pharmacological interventions; however, they failed to show the repeatability of positive outcomes. Moreover, there is consensus for the complexity of the physical illness of CFS/ME, as evidenced by accumulating scientific findings [1]. Accordingly, to explore the pathophysiology of CFS/ME, new systematic research strategies are essential for developing fundamental treatments, especially for pharmaceutical interventions, although most drug-based RCTs have failed so far.

Our review has some limitations. We searched literatures from PubMed and Cochrane library. Although these two databases are the major resources for scientific articles especially derived from RCTs, there would be a possibility of further information in other databases. In order to produce confident data, we excluded the too small-scale RCTs (< 45 participants), however this strategy also has a risk to loss any valuable information. In addition, only 9 of 55 RCTs had presented fragmentary data related to blood parameters. We could hardly find any practical indications due to very heterogenous parameters and no significant correlation with changes of fatigue symptoms. The identification of the blood-based biomarkers is necessary for diagnosis as well as classification of CFS/ME and should be applied to clinical trials in the future. Nevertheless, this is the first systematic review of RCTs targeting CFS/ME regardless of language, and this review shows the comprehensive features of CFS/ME. Our review offers fundamental information for future research on the pathophysiology of and new treatments for CFS/ME.

## Conclusion

This systematic review provides a comprehensive integration of previous RCTs for CFS/ME. Our data include characteristics of RCTs such as participants, case definitions, interventions and primary measurements. In addition, we found trends in the interventions used as well as in the overall results. Psychological treatments were predominant and had limitations curing CFS/ME. An exploration of the pathophysiology of CFS/ME and better development of treatments are needed.

## Data Availability

All data analyzed during this study are available in the public domain.

## Abbreviations

BDI: Beck Depression Inventory
CBT: cognitive behavior therapy
CFS: chronic fatigue syndrome
CGI: Clinical Global Impression
CHQ (-CF): Child Health Questionnaire (-Child Form)
CIS: Checklist Individual Strength
CPRS: Comprehensive Psychopathological Rating Scale
DLE: dialyzable leukocyte extract
FIS: Fatigue Impact Scale
FITNET: Fatigue in Teenagers on the interNET
FSM:ACT: Fatigue Self-Management with web diaries and actigraphs
FSM:CTR: Fatigue Self-Management with paper diaries and pedometers
FSS: Fatigue Severity Scale
GET: graded exercise therapy
GHQ: General Health Questionnaire
HAMD: Hamilton rating scale for Depression
HC: healthy control
HRQL: health-related quality of life
iCBT: Internet-based CBT
KPS: Karnofsky Performance Score
MFI: Multidimensional Fatigue Inventory
MFS: Mental Fatigue Scale
MRT: multidisciplinary rehabilitation treatment
POMS: Profile of Mood States
QoL: quality of life
SAR: school attendance rate
SAS: Self-rating Anxiety Scale
SF-36: 36-item Short Form health survey
SIP: Sickness Impact Profile
MC: medical care
VAS: Visual Analogue Scale
