# Correction to: Systematic review of randomized controlled trials for chronic fatigue syndrome/myalgic encephalomyelitis (CFS/ME)

**DOI:** 10.1186/s12967-020-02603-6

**Published:** 2020-12-23

**Authors:** Do-Young Kim, Jin-Seok Lee, Samuel-Young Park, Soo-Jin Kim, Chang-Gue Son

**Affiliations:** 1 Korean Medical College of Daejeon University, 62, Daehak-ro, Dong-gu, Daejeon, Republic of Korea; 2 Institute of Traditional Medicine and Bioscience, Dunsan Oriental Hospital of Daejeon University, 75, Daedeok-daero 176, Seo-gu, Daejeon, Republic of Korea

## Correction to: J Transl Med (2020) 18:7. 10.1186/s12967-019-02196-9

Following publication of the original article [1], the authors noted that one study (PACE trial) [2] had been missed in the captured data. Accordingly, some corrections were made in multiple sections, including the addition of the reference information for the PACE trial in the reference list. The updated sections are given in this Correction, and the changes have been highlighted in **bold typeface.** The original article [1] has been corrected.

## Abstract

The updated sentences are given below, and the changes have been highlighted in **bold typeface**.

**Result:** Among 513 potentially relevant articles, **56** RCTs met our inclusion criteria; these included 25 RCTs of 22 different pharmacological interventions, **29** RCTs of **19** non-pharmacological interventions and 2 RCTs of combined interventions. These studies accounted for a total of **6956** participants (**1713** males and **5243** females, **6499** adults and 457 adolescents). CDC 1994 (Fukuda) criteria were mostly used for case definitions (42 RCTs, **75.0**%), and the primary measurement tools included the Checklist Individual Strength (CIS, **35.7**%) and the 36-item Short Form health survey (SF-36, **32.1**%). Eight interventions showed statistical significance: 3 pharmacological (Staphypan Berna, Poly(I):poly(C12U) and CoQ10 + NADH) and 5 non-pharmacological therapies (cognitive-behavior-therapy-related treatments, graded-exercise-related therapies, rehabilitation, acupuncture and abdominal tuina). However, there was no definitely effective intervention with coherence and reproducibility.

## Result section

The updated sentences are given below, and the changes have been highlighted in **bold typeface**.

### Characteristics of RCTs meeting the inclusion criteria

From the PubMed and Cochran databases, a total of 513 articles were initially identified, and **56** articles ultimately met the inclusion criteria for this study (Fig. 1). Fifty-**one** RCTs (**91.1**%) were conducted for adult patients, while 5 RCTs (**8.9**%) were conducted for the adolescent population (Table 1). The majority of RCTs were conducted in 3 countries: the UK (n = **16**), the Netherlands (n = 14), and the USA (n = 9). Regarding interventions, **29** RCTs (**51.8**%) conducted nonpharmacological interventions, 25 RCTs (**44.6**%) conducted pharmacological interventions and 2 RCTs conducted a combination of pharmacological and nonpharmacological interventions (Tables 2 and 3).

### Characteristics of participants and case definitions for inclusion criteria

In **56** RCTs, a total of **6956** participants (**1713** males and **5243** females, **6499** adults with a mean age of **40.2 ± 4.0** years and 457 adolescents with a mean age of 15.5 ± 0.3 years) were enrolled. Fifty-**five** RCTs (98.2%) adapted at least one of the following CFS case definitions: CDC 1994 (Fukuda) criteria (42 RCTs), Oxford 1991 (Sharpe) criteria (**13** RCTs), CDC 1988 (Holmes) criteria (3 RCTs), Lloyd 1988 criteria (2 RCTs), and Schluederberg 1992 (2 RCTs).

### Main outcome measurement

A total of 31 primary measurement tools were used to assess the main outcome in **56** RCTs. The Checklist Individual Strength (CIS) was the most frequently used (**35.7**%), and others included the 36-item Short Form health survey (SF-36, **32.1**%), Sickness Impact Profile (SIP, **14.3**%), Chalder Fatigue Scale (**14.3**%), Visual Analogue Scale (VAS, **10.7**%) and Clinical Global Impression (CGI, **8.9**%). There were **29** RCTs that used multiple primary measurements (Table 1).

### RCTs with nonpharmacological interventions

There were **29** RCTs in the nonpharmacological category (**26** for adults, 3 for adolescents) with **19** kinds of interventions, mainly CBT (n = **12**), exercise (n = **6**), and self-care (n = 5). The mean treatment period was **18.5 ± 8.9** weeks (**17.1 ± 7.1** weeks for adults, 30.7 ± 15.1 weeks for adolescents). Of the **12** CBT subcategories, **6** RCTs showed statistical effectiveness of CBT compared to the control [41, 44, **46, 49, 50, 52**]. In addition, **4** RCTs of graded-exercise-related therapies [**46, 53, 55, 56**] and 3 RCTs of integrative, consumer-driven rehabilitation [**64**], acupuncture [**65**] and abdominal tuina [**67**] showed a significantly effect of the intervention compared to the control (Table 3).

## Discussion section

The updated sentences are given below, and the changes have been highlighted in **bold typeface.** Sentences with only a change in reference citations numbering (the original references 46–92 were re-numbered to 47–93) are not provided.

### The first paragraph (the 3rd sentence)

To support future studies for CFS/ME treatments, we systematically reviewed **56** RCTs to investigate characteristics such as participants, case definitions, interventions and primary measurements.

### The second paragraph (the 1st sentence)

The sex ratio of the participants was male 1 vs. female 3 (**1713/5143**, except one RCT had recruited only females).

### The third paragraph (the 1st–4th sentences)

A total of **56** RCTs included 25 pharmacological, **29** nonpharmacological and 2 combined interventions (Table 1). The mean treatment period of the RCTs with nonpharmacological interventions was longer than that with medication, especially for adolescents (total: **18.5 ± 8.9** vs. 10.8 ± 6.8, adolescent: 30.7 ± 15.1 vs. 8.5 ± 0.7, Table 1). Periodically, the trials gradually increased, with 13 trials in the 1990s, 19 trials in the 2000s and **24** trials in the 2010s. The pharmacological RCTs were predominant in the 1990s and 2000s, while nonpharmacological interventions became predominant in the 2010s (pharmacological:nonpharmacological ratio from 20:14 to 7:**17**) (data not shown).

### The fifth paragraph (the 8th and 11th sentences)

Contrary to the positive outcomes in the 1990s and 2000s, more recent CBT trials have failed to show consistent benefits in patients with CFS/ME: 5 of **8** RCTs of CBT did not show significant effects in our data.

In our data, **5** of **6** RCTs with graded-exercise-related therapies presented positive outcomes; however, the clinical usefulness of GET is highly controversial [**89**].

### The eighth paragraph (the 5th sentence)

In addition, only 9 of **56** RCTs had presented fragmentary data related to blood parameters.

## Reference section

As one RCT (PACE trial) was added, its reference information [2] was included in the reference list as reference number 46. Accordingly, the original references 46–92 were re-numbered to 47–93.

## Figures

Figures 1 and 2.

**Fig. 1:**
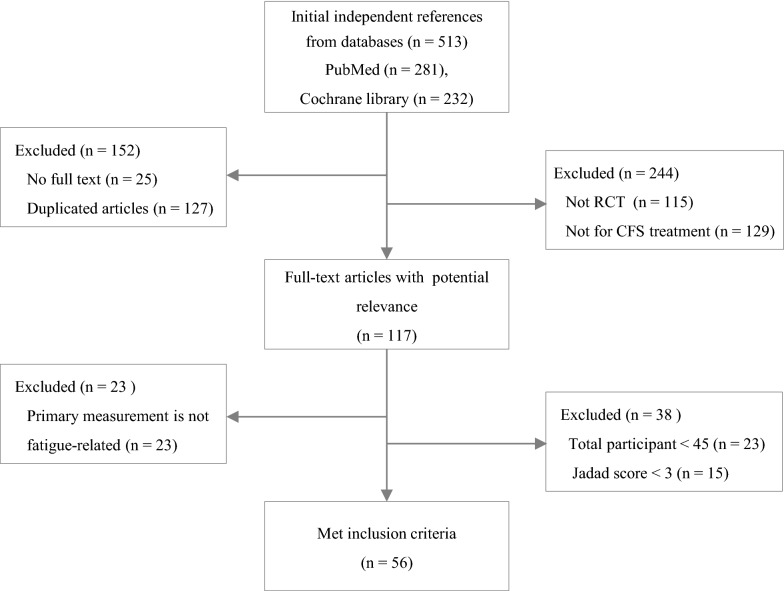
Flow chart of study. The numbers of literatures were changed and highlighted in **bold typeface.** ‘Excluded (n= **244**)’ on upper-right box, ‘Not RCT (n = **115**)’, ‘Not for CFS treatment (n = **129**)’, ‘Full-text articles with potential relevance (n = **117**)’ and ‘Met inclusion criteria (n= **56**)’

**Fig. 2:**
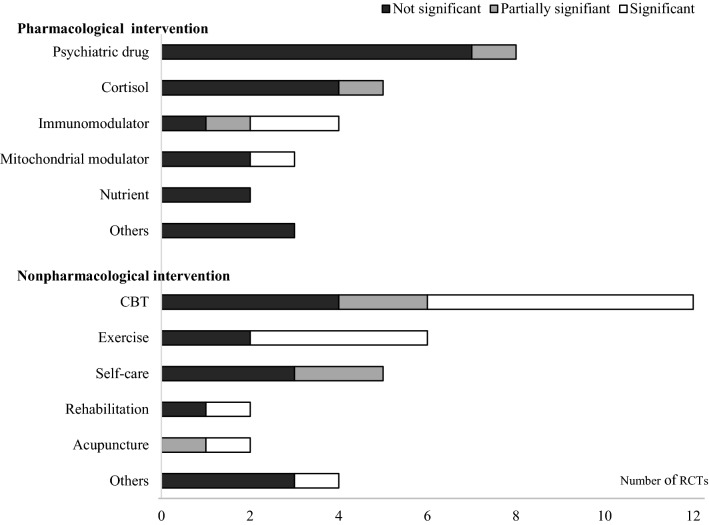
Graphical display for statistical significance of interventions. Number of RCTs in nonpharmacological intervention were added one each in CBT (significant), Exercise (significant) and others (not significant)

## Tables

The updated Tables 1, 3 and 4 are given below, and the changes have been highlighted in **bold typeface.**

**Table 1:** Study characteristics

Items	Adults	Adolescents	Total
N. of RCT (%)	**51 (91.1)**	5 **(8.9)**	**56** (100.0)
N. of participants (%) (males/females)	**6499 (93.4) (1611/4888)**	457 **(6.6)** (102/355)	**6956** (100.0) **(1713/5243)**
Mean N. of participants	**127.4 ± 113.3**	91.4 ± 33.5	**124.2 ± 109.0**
Mean age (year)^a^	**40.2 ± 4.0**	15.5 ± 0.3	**38.7 ± 8.1**
N. of case definitions for inclusion criteria (%)^b,c^			
CDC 1994 (Fukuda)	37 **(72.5)**	5 (100.0)	42 **(75.0)**
Schluederberg 1992	2 **(3.9) 12 (23.5)**	–	2 (3.6) **13 (23.2)**
Oxford 1991 (Sharpe)	3 **(5.9)**	1 (20.0)	3 **(5.4)**
CDC 1988 (Holmes)	2 **(3.9)**	–	2 (3.6)
Lloyd 1988	5 **(9.8)**	–	6 **(10.7)**
Others		1 (20.0)	
RCTs with pharmacological intervention (N, %)	23 (92.0)	2 (8.0)	25 (100.0)
Kinds of interventions (%)	20 (90.9)	2 (9.1)	22 (100.0)
Mean treatment period (weeks)	11.0 ± 7.0	8.5 ± 0.7	10.8 ± 6.8
RCTs with nonpharmacological intervention (N, %)	**26 (89.7)**	3 **(10.3)**	**29** (100.0)
Kinds of interventions^d^	**18 (94.7)**	2 **(10.5)**	**19** (100.0)
Mean treatment period (weeks)	**17.1 ± 7.1**	30.7 ± 15.1	**18.5 ± 8.9**
RCTs with combined interventions (N, %)	2 (100.0)	–	2 (100.0)
Kinds of interventions (%)	4 (100.0)	–	4 (100.0)
Mean treatment period (weeks)	26 ± 2.8	–	26 ± 2.8
Primary measurements in 55 RCTs (n, %)^c,e^			
Checklist Individual Strength (CIS)		20 **(35.7)**	
36-item Short Form health survey (SF-36)		**18 (32.1)**	
Sickness Impact Profile (SIP)		8 **(14.3)**	
Chalder Fatigue Scale		**8 (14.3)**	
Visual Analogue Scale (VAS)		6 **(10.7)**	
Clinical Global Impression (CGI)		5 **(8.9)**	
Karnofsky Performance Scale (KPS)		3 **(5.4)**	
School attendance rate (SAR)		3 **(5.4)**	
Multidimensional Fatigue Inventory (MFI)		2 (3.6)	
Fatigue Severity Scale (FSS)		2 (3.6)	
Others		21 **(37.5)**	

^a^This is the mean of ages presented as median or mean in original articles

^b^Twelve RCTs used two case definitions for inclusion criteria

^c^Some items have been applied multiple times, thus the total percentage is larger than 100%

^d^One intervention (CBT) was used for both of adult and adolescent studies

^e^Twenty**-nine** RCTs used multiple primary measurements

**Table 3:** RCTs with nonpharmacological interventions

Intervention	N. of participants (N. of arms, control)	Period (week)	Primary measurement (subscale)	Significance
**CBT**				
iCBT [41]	240 (3, waitlist)	27	CIS (fatigue)	P < 0.01
Group CBT [42]	204 (3, waitlist)	24	CIS (fatigue), SF-36 (physical score)	CIS: d > 0.8
CBT [43]	122 (2, MRT)	24	CIS (fatigue), SF-36	Not significant
FITNET [44]	135 (2, usual care)	48	SAR, CIS (fatigue), CHQ (physical score)	P < 0.01
CBT + GET [46]	120 (2, usual care)	24	SF-36	Not significant
**CBT** [46]	**640 (4, MC)**	**24**	**Chalder scale, SF-36 (physical score)**	**P < 0.01**
Family-focused CBT [**47**]	63 (2, psychoeducation)	24	SAR	Not significant
Group CBT [**48**]	153 (3, education + support, MC)	16	SF-36 (physical, mental score)	Not significant
CBT [**49**]	71 (2, waitlist)	20	CIS (fatigue), SF-36 (physical score), SAR	CIS, SF-36: P < 0.01, SAR: P < 0.05
CBT [**50**]	278 (3, guided support, no treatment)	32	CIS (fatigue), SIP-8	CIS: P < 0.01, SIP: P < 0.05
CBT [**51**]	60 (2, relaxation)	16–24	Chalder scale, SF-36 (physical score)	Chalder scale: P < 0.01
CBT [**52**]	60 (2, MC)	16	Karnofsky normal function scale	P < 0.01
**Exercise**				
Guided exercise self-help [**53**]	211 (2, MC)	12	Chalder scale, SF-36 (physical score)	P < 0.01
Qigong [**54**]	64 (2, waitlist)	16	Chalder scale, SF-12	Not significant
**GET** [**46**]	**640 (4, MC)**	**24**	**Chalder scale, SF-36 (physical score)**	**P < 0.01**
GET [**55**]	49 (2, MC)	12	Self-rated global change score	P < 0.05
Education to encourage graded exercise [**56**]	148 (4, MC)	16	SF-36 (physical score)	P < 0.01
Graded aerobic exercise [**57**]	66 (crossover, flexibility therapy)	12	CGI	Not significant
**Self-care**				
Fatigue self-management [**58**]	137 (3, usual care)	12	FSS	Not significant
Group-based self-management [**59**]	137 (2, usual care)	16	SF-36 (physical score)	Not significant
Guided self-instruction [**60**]	123 (2, waitlist)	20	CIS (fatigue), SF-36 (physical, social score)	CIS: P < 0.01
Stepped care [**61**]	171 (2, CBT)	16	CIS (fatigue), SIP-8, SF-36 (physical score)	Not significant
Guided self-instruction [**62**]	169 (2, waitlist)	16	CIS (fatigue), SIP-8, SF-36 (physical score)	CIS, SIP8: P < 0.01
**Rehabilitation**				
Pragmatic rehabilitation [**63**]	302 (3, supportive listening, general treatment)	18	Chalder scale, SF-36 (physical score)	Not significant
Integrative, consumer-driven rehabilitation [**64**]	47 (2, delayed program)	16	CFS Symptom Rating Form, The QoL Index	P < 0.05
**Acupuncture**				
Acupuncture [**65**]	150 (3, sa-am, no treat)		FSS	P < 0.05
Acupuncture [**66**]	100 (2, sham)		Chalder scale, SF-12, GHQ-12 (mental score)	Chalder scale: P < 0.05
**Others**				
Abdominal tuina [**67**]	77 (2, acupuncture)		Chalder scale, SAS, HAMD	P < 0.05
**Adaptive pacing** [**46**]	**640 (4, MC)**	**24**	**Chalder scale, SF-36 (physical score)**	**Not significant**
Low-sugar, low-yeast diet [**68**]	52 (2, healthy eating)	24	Chalder scale, SF-36	Not significant
Distant healing [**69**]	409 (4, not knowing, no treat)	24	SF-36 (mental score)	Not significant

*CBT* cognitive behavior therapy, *FITNET*: Fatigue In Teenagers on the interNET, *GET* graded exercise therapy, *CIS* Checklist Individual Strength, *SF-36* 36-item Short Form health survey, *SAR* school attendance rate, *CHQ* Child Health Questionnaire, *SIP-8* Sickness Impact Profile, *CGI* Clinical Global Impression, *FSS* Fatigue Severity Scale, *GHQ-12* General Health Questionnaire-12, *SAS* Self-rating Anxiety Scale, *HAMD* Hamilton rating scale for Depression

**Table 4:** RCTs with pharmacological and nonpharmacological combined interventions

Intervention	Intervention	Intervention	Intervention	Intervention
Fluoxetine + graded exercise [**70**]	Exercise + fluoxetine: 33 Exercise + placebo: 34 Appointment + fluoxetine: 35 Appointment + placebo: 34	24 20 mg/day	Chalder scale	Graded exercise P < 0.05
Dialyzable leukocyte extract (DLE) + CBT [**71**]	DLE + CBT: 20 DLE + clinic: 26 Placebo + CBT: 21 Placebo + clinic: 23	28 5 × 10^8^ leukocytes 8 times biweekly	VAS (global well-being)	Not significant

*VAS* Visual Analogue Scale
